# What Factors Affect the Evolution of the Wife’s Mental Health After the Husband’s Retirement? Evidence From a Population-Based Nationwide Survey in Japan

**DOI:** 10.2188/jea.JE20200071

**Published:** 2021-05-05

**Authors:** Takashi Oshio

**Affiliations:** Institute of Economic Research, Hitotsubashi University, Tokyo, Japan

**Keywords:** marital quality, mental health, random-effects model, retired husband syndrome, social participation

## Abstract

**Background:**

The “retired husband syndrome” refers to the negative impact of the husband’s retirement on the wife’s health. This study provided new insights by examining whether and to what extent the wife’s social participation, interactions with her husband, and job status prior to her husband’s retirement affected the evolution of her mental health after her husband’s retirement.

**Methods:**

We collected data from a 12-wave nationwide panel survey conducted from 2005 to 2016, starting with individuals aged 50–59 years. Focusing on 3,794 female respondents whose husbands retired during the survey period, we applied random-effects linear regression models to investigate the evolution of their mental health as measured by the Kessler 6 (K6) score (range, 0–24; Mean, 3.41; standard deviation, 4.11) during the 5 years after their husbands’ retirement.

**Results:**

On average, the wife’s K6 score rose by 0.18 (95% confidence interval [CI], 0.08–0.28), 0.18 (95% CI, 0.03–0.34), and 0.19 (95% CI, −0.02 to 0.43) in the first 3 years, respectively, after the husband’s retirement, before declining toward the baseline level. However, the wife’s active social participation, intense interactions with her husband, and absence of paid employment before her husband’s retirement prevented her mental health from deteriorating.

**Conclusion:**

The results suggest the limited relevance of the “retired husband syndrome” among middle-aged Japanese couples. The effects of a husband’s retirement on the wife’s mental health depended heavily on her prior behavior.

## INTRODUCTION

Retirement affects individuals’ physical and mental health as well as health behavior,^1^^–^^8^ because it represents a key transition in lifestyle for middle-aged individuals. In recent years, increasing attention has been paid to the impact of an individual’s retirement on his/her spouse’s health.^9^^–^^12^ Specifically, studies have examined the existence of what is called the “retired husband syndrome,”^9^ whereby the husband’s retirement has a negative impact on the wife’s health outcomes, such as increased stress or depression and worsened physical health. A change in the husband’s daily lifestyle after retirement—for instance, from regularly going out every weekday to staying home almost every day—likely increases the psychological pressure on his wife and negatively impacts other aspects of her health, especially if the transition to retirement did not proceed smoothly. A failure of a smooth transition of the husband’s daily lifestyle may also increase his physical demand for his wife’s homemaking tasks, which in turn will increase her mental strain, as suggested by the theories of effort-reward imbalances^13^ and demand-control (-support) models^14^^,^^15^ in occupational health.

However, previous observations regarding retired husband syndrome have not been fully consistent. For example, one study found a negative impact of the husband’s retirement on the wife’s mental health outcomes, such as stress, depression, and inability to sleep among married Japanese women.^10^ Similarly, a cross-country analysis based on data from 19 European countries revealed that the husband’s retirement had a negative effect on the wife’s subjective health status.^11^ In contrast, one recent study based on data from a Chinese social survey showed that the husband’s retirement improved the wife’s physical and mental well-being.^12^

These mixed observations seem to be attributable to several factors. From a life course perspective, adaptation to life transitions depends on not only the occurrence of the transition per se but also the specific context in which the transition takes place.^16^ In the case of retirement, the couple’s pre-retirement conditions may affect their post-retirement well-being, and their subjective assessment of adaptation to retirement may be complicated by gender role ideology and other social norms. In addition, cross-sectional analysis may fail to identify causation from retirement to health, which is another possible reason for the mixed relationships that have been found.

Based on a longitudinal dataset of married couples, the current study focused on the effect modification of the wife’s behavior prior to her husband’s retirement for this syndrome; that is, it investigated whether and to what extent the wife’s behavior prior to her husband’s retirement affected the evolution of her mental health afterwards. This issue, which has been largely understudied, is expected to gain importance over time, especially in Japan because ongoing policy reforms related to mandatory retirement and public pension programs^17^ might affect married couples’ post-retirement health and well-being.

This study specifically focused on three aspects of the wife’s behavior prior to her husband’s retirement: (1) social participation, (2) interactions with her husband, and (3) job status. We examined how these aspects would modify the effect of the husband’s retirement on the wife’s mental health, following a study that stressed the importance of pre-retirement influences on post-retirement subjective well-being.^18^ Related to (1) social participation, studies have shown that social participation can mitigate the shocks to mental health caused by risk events such as the onset of a diagnosed disease and caregiving for elderly parents.^16^^,^^17^ Hence, it is reasonable to predict that the wife’s social participation would mitigate the adverse impact, if any, of her husband’s retirement on her mental health. As for (2) husband-wife interactions, studies have shown that high marital quality can contribute to the couple’s post-retirement well-being.^18^^,^^21^ A wife who has enjoyed joint activities with her husband is predicted to be able to adjust reasonably smoothly to life with her retired husband. However, it is less obvious how (3) the wife’s job status may modify the impact of her husband’s retirement on her mental health. As long as the husband is employed, his wife may carry the main responsibility for housework even if she is employed. However, she may no longer accept doing so after her husband’s retirement and hence feel stressed if he does not take over housework.^13^ If that is the case, the wife’s employment is expected to add to the negative impact of her husband’s retirement on her mental health. We cannot exclude the opposite possibility, nevertheless, depending on socio-cultural contexts and other factors.^22^^,^^23^

## METHODS

### Study sample

We used data obtained from a nationwide 12-wave panel survey, “The Longitudinal Survey of Middle-Aged and Older Adults,” conducted by the Japanese Ministry of Health, Labour and Welfare (MHLW) each year from 2005 to 2016. Japan’s Statistics Law required the survey to be reviewed from statistical, legal, ethical, and other viewpoints. We obtained the survey data from the MHLW with its official permission; therefore, the current study did not require ethical approval.

The sample in the first wave was limited to those aged 50–59 years. This nationwide sample was selected in November 2005 through a two-stage random sampling procedure. A total of 34,240 individuals responded (response rate: 83.8%). The second to tenth waves of the survey were conducted in early November of each year from 2006 to 2016, and 21,916 individuals remained in the tenth wave (average attrition rate of 4.0% in each wave). No new respondents were added after the first wave.

We focused on married female respondents whose husbands were observed to have retired in a certain wave between the second and twelfth waves. We defined retirement as the onset of the transition from the period during which one earned wage income—whether as a full- or part-time worker—to the period during which one earned no wage income. Retirement as defined here was closely linked to but not necessarily equivalent to the mandatory retirement age or the eligibility age for claiming public pension benefits. We did not include data after the husbands resumed working, and assumed that if the husbands had worked in the first wave (2005), they had been working continuously until then.

**Figure 1.**
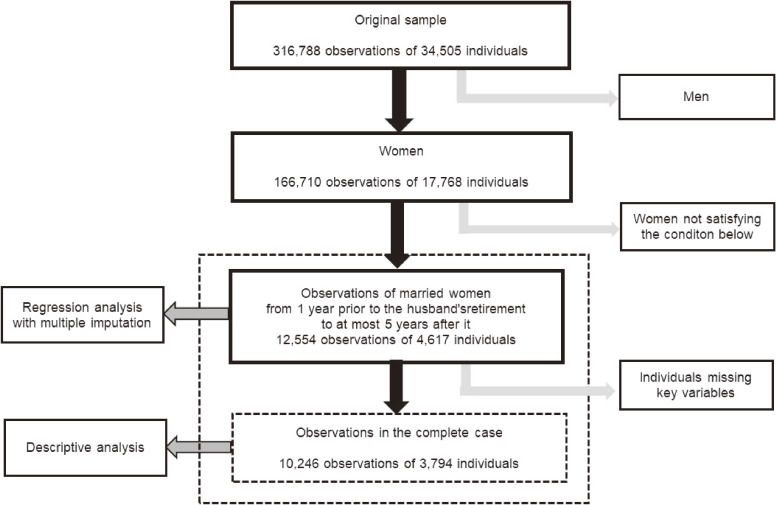
Construction of the study sample

For statistical analysis, we used the data of 12,554 observations of 4,617 married women observed from 1 year prior to the husband’s retirement to at most 5 years after it. We concentrated on the first 5 years after the husband’s retirement in light of the possibility that other factors might affect the evolution of the wife’s mental health when an extended time passed after her husband’s retirement.^16^ After excluding those missing key variables, we used 10,246 observations of 3,794 married women for descriptive analysis. For regression analysis, we adopted the multiple imputation approach to mitigate potential biases caused by missing information. We generated 10 imputed datasets using multiple imputation under the chained equations procedure, and used them for regression analysis. Figure 1 summarizes how we constructed the study sample.

### Measures

We used the Kessler 6 (K6) score to measure psychological distress.^24^^,^^25^ Earlier studies have confirmed the reliability and validity of this score in psychological analyses of Japanese people.^26^^,^^27^ In the present study, the respondents were required to answer the following six-item psychological distress questionnaire: “During the past 30 days, how often did you feel: a) nervous; b) hopeless; c) restless or fidgety; d) so depressed that nothing could cheer you up; e) that everything was an effort; and f) worthless?” This was rated on a five-point Likert scale (0 = *none of the time* to 4 = *all of the time*). We then calculated the sum of the reported scores (range: 0–24) and defined it as the K6 score. Cronbach’s alpha was 0.897 for this study sample. Higher K6 scores reflect higher levels of psychological distress.

Regarding social participation, the survey asked the respondents whether they participated in each of six types of social activity: “hobbies or cultural activities,” “exercise or sports,” “community events,” “support for children,” “support for the elderly,” and “other activities.” We constructed a binary variable for “active” social participation by allocating one point to the respondents who reported participating in three or more of these six types. As for interactions with the husband, the survey asked the respondents whether they did each of eight activities when spending time together with their spouses: “chatting,” “hobbies or cultural activities,” “shopping,” “voluntary activities,” “job,” “dining,” “watching TV,” and “other.” We constructed a binary variable for “intensive” interactions with the husband by allocating one point to the respondents who reported participating in five or more of these eight types. For wife’s job status, we constructed a binary variable of “doing a paid job” by allocating one point to those who reported they were engaged in paid work.

As for covariates, we first considered educational attainment (categorized as junior high school, high school, junior college, college or above, and other) and age (years) for both wives and husbands. We also considered the baseline conditions 1 year prior to the husbands’ retirement in terms of their wives’ scores of self-rated health (ordinal variable scored 1 = *excellent*, 2 = *good*, 3 = *somewhat good*, 4 = *somewhat poor*, 5 = *poor*, and 6 = *very poor*). As a proxy for household income, we also considered household spending adjusted for household size by dividing it by the square root of the number of household members.^28^^,^^29^

### Analytic strategy

Following a descriptive analysis to summarize the key features of the respondents used in the statistical analysis, we estimated random-effects linear regression models to explain the changes in a wife’s K6 scores from its level 1 year prior to her husband’s retirement. First, to capture the overall picture of the evolution of the wife’s mental health after her husband’s retirement, we estimated the benchmark model:

$$\Delta k_{\text{ti}}=\sum_{t=1}^{5}\alpha_{t}{\text{year}}_{\text{ti}}+(\text{covariates})+e_{i}+\varepsilon_{\text{ti}},$$

for the *i*-th respondent in the *t*-th year after her husband’s retirement (with *t* = 1 indicating the onset of retirement). Δ*k* indicates a change in K6 score from 1 year prior to the husband’s retirement, *year* is a binary indicator for each year after retirement (taking 1 year prior to retirement as reference), *e* represents each respondent’s fixed effects, and ε is an error term. The estimated coefficients (α) for each year are expected to have a positive sign if the husband’s retirement has an adverse impact on the wife’s mental health.

We then added a binary variable of the wife’s behavior prior to her husband’s retirement and its interaction terms with the binary variables of each year to the benchmark model:

$$\begin{array}{rl} \Delta k_{\text{ti}} & =\sum_{t=1}^{5}(\beta_{t}{\text{year}}_{\text{ti}}+\beta^{\prime }{}_{t}X_{i}\times {\text{year}}_{\text{ti}})+\delta X_{i}\\ & \;+(\text{covariates})+e_{i}+\varepsilon_{\text{ti}}, \end{array}$$

where *X* is a binary variable of the wife’s behavior corresponding to the wife’s active social participation, intense interactions with her husband, or doing a paid job. We compared the estimated values of β and β + β′, the latter of which was computed after regression, along with their statistical significance, to assess the impact of the wife’s behavior on the evolution of her mental health after her husband retired.

We further conducted a supplementary analysis to deal with the potential endogeneity of the husband’s retirement, based on an instrumental variable (IV) approach using public pension eligibility as an instrument, following many preceding studies.^2^^,^^3^^,^^10^^,^^11^ Specifically, we first estimated the linear regression model to predict the husband’s retirement age by the eligibility ages for claiming the flat and wage-proportional benefits of the Employees’ Pension Insurance Program,^30^ corresponding to his cohort—for instance, these eligibility ages are 62 and 65 years, respectively, for men born from April 1955 to March 1957—along with covariates. We then took the estimated (and rounded) retirement age as the baseline, correspondingly arranged other variables related to it, and re-estimated the regression models to explain the changes of the wife’s K6 score after the estimated retirement age. Unlike conventional IV, this approach needs manual adjustments but is expected to help mitigate potential endogeneity problems. We used STATA 16 (Stata Corp., College Station, TX, USA) for all analyses.

## RESULTS

Table 1 summarizes the key features of the respondents observed 1 year prior to the husband’s retirement for all female respondents as well as each group divided by the wife’s behavior. The wives who were active in social participation, had intensive interactions with their husbands, and performed paid work made up 28.4%, 37.1%, and 59.6%, respectively, of the entire sample. The average husband’s retirement age was slightly above 60 years in all groups, probably reflecting that the mandatory retirement was set at age 60 for the study cohorts. The wives were 2.3 to 2.4 years younger than their husbands. Both the K6 and self-rated health scores were somewhat lower (better) among wives who were active in social participation, had intensive interactions with their husbands, and did a paid job than others.

**Table 1.** Key features of the study sample

Wife’s behavior		All	Social participation		Interactions with the husband		Doing a paid job	

			Inactive	Active	Not intensive	Intensive	No	Yes
Wife								
K6 score^a^ (range, 0–24)	*M*	3.36	3.55	2.80	3.54	2.94	3.61	3.19
	*SD*	(4.11)	(4.28)	(3.48)	(4.29)	(3.63)	(4.37)	(3.91)
Self-rated health^a^ (range, 1–6)	*M*	2.77	2.82	2.62	2.81	2.69	2.88	2.69
	*SD*	(0.86)	(0.88)	(0.81)	(0.87)	(0.85)	(0.93)	(0.81)
Age	*M*	58.3	58.0	59.0	58.0	58.3	59.1	57.7
	*SD*	(3.6)	(3.5)	(3.6)	(3.6)	(3.6)	(3.5)	(3.5)
Graduated from college or above (%)		7.0	6.4	9.0	6.1	8.6	7.6	6.6

Husband								
Age	*M*	60.6	60.3	61.5	60.4	60.7	61.5	60.0
	*SD*	(4.0)	(4.0)	(4.0)	(4.1)	(4.0)	(3.9)	(4.0)
Graduated from college or above (%)		26.5	24.3	34.1	24.4	30.2	30.3	23.9
Household spending^b^ (monthly, thousand JPY)	*M*	183.9	179.2	199.3	177.3	195.9	186.0	182.4
	*SD*	(137.6)	(110.8)	(191.7)	(106.7)	(188.5)	(105.8)	(155.6)

*N*		3,794	2,560	1,015	2,120	1,250	1,531	2,260
Proportion (%)		(100.0)	(71.6)	(28.4)	(62.9)	(37.1)	(40.4)	(59.6)

JPY, Japanese yen; K6, Kessler Screening Scale for Psychological Distress; SD, standard deviation.

^a^The higher, the worse.

^b^Adjusted household size.

Table 2 presents the estimation results of the benchmark regression model, which aimed to capture the overall evolution of the wife’s K6 score after her husband’s retirement, after the mutual imputation procedure. The K6 score initially rose by 0.18 (95% confidence interval [CI], 0.08–0.28), which was equivalent to approximately 4% of one standard deviation of the score, in response to the husband’s retirement. The K6 score remained above the level prior to the husband’s retirement until the second year, and then the difference from the pre-retirement level turned non-significant. We confirmed that this pattern of evolution remained largely intact even when including data after the fifth post-retirement year (not reported in the table). As for other variables, self-rated health scores at baseline reduced the rise in post-retirement K6 score, while other covariates were not related to it.

**Table 2.** The estimation results of the random-effects regression model explaining the change in the wife’s K6 score from the level 1 year prior to her husband’s retirement^a^

	Coef. (α)	95% CI
After the husband’s retirement		
1st year	0.18^***^	(0.08, 0.28)
2nd year	0.18^*^	(0.03, 0.34)
3rd year	0.19^†^	(−0.02, 0.39)
4th year	0.10	(−0.14, 0.41)
5th year	−0.06	(−0.34, 0.24)
Self-rated health at baseline	−0.24^***^	(−0.30, −0.18)
Age	−0.01	(−0.03, 0.02)
Husband’s age	0.00	(−0.02, 0.02)
Educational attainment		
High school	−0.14	(−0.31, 0.03)
Junior college	−0.19	(−0.42, 0.05)
College or above	−0.24	(−0.52, 0.04)
Other	0.55	(−0.32, 1.42)
Husband’s educational attainment		
High school	0.03	(−0.13, 0.19)
Junior college	−0.10	(−0.46, 0.27)
College or above	−0.10	(−0.30, 0.10)
Other	0.04	(−0.55, 0.62)
Household spending at baseline^b^	−0.12	(−0.05, 0.02)

CI, confidence interval.

^a^12,554 observations of 12,554 individuals after multiple imputation.

^b^Household-size adjusted, monthly, million Japanese yen.

^***^P < 0.001, ^**^P < 0.01, ^*^P < 0.05.

**Figure 2.**
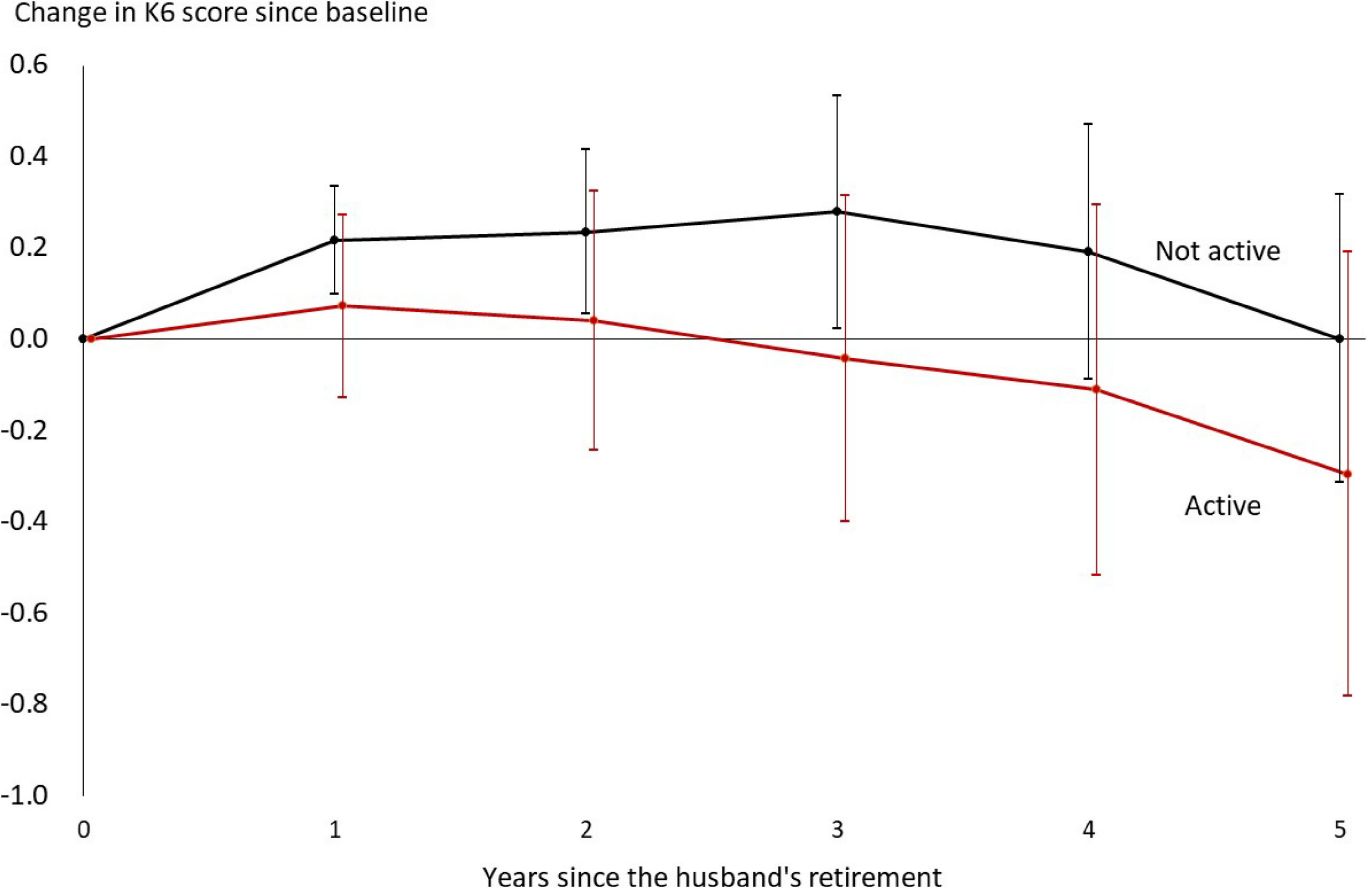
Comparing the evolution of K6 scores after the husband’s retirement between wives who were active in social participation at baseline and those who were not^a^ ^a^Means and 95% confidence intervals are indicated, based on the first panel of Table 3.

Table 3 shows how the evolution of the K6 score depended on the wife’s behavior before the husband’s retirement. In the case of social participation, the K6 score deteriorated among inactive respondents; the score exceeded the baseline level in the first year and remained above it until the third year. In contrast, the score did not deteriorate at all among active respondents. The former result seems to have dominated the evolution of the K6 score for the entire sample reported in Table 2. We graphically illustrate these results in Figure 2, in which we compare the paths of the changes in the K6 score from the baseline between two types of female respondents. As suggested by the overlapping 95% CI bands, the difference in the two paths was not statistically significant. However, the figure confirms that mental health worsened only among those without active social participation. We obtained similar results for the interactions with the husband and job status. As seen in Table 3, mental health did not deteriorate among the respondents who had intensive interactions with their husbands or those who did not work.

**Table 3.** Comparing the changes in the wife’s K6 score by her baseline behavior^a,b^

	Coef. (β)	95% CI	Coef. (β + β′)	95% CI
Social participation	Less active		Active	
1st year	0.22^***^	(0.10, 0.34)	0.07	(−0.13, 0.27)
2nd year	0.24^*^	(0.05, 0.42)	0.04	(−0.24, 0.33)
3rd year	0.28^*^	(0.02, 0.53)	−0.04	(−0.40, 0.31)
4th year	0.19	(−0.09, 0.47)	−0.11	(−0.52, 0.29)
5th year	0.00	(−0.31, 0.32)	−0.30	(−0.78, 0.19)

Interactions with the husband	Less intensive		Intensive	
1st year	0.22^***^	(0.10, 0.35)	0.10	(−0.08, 0.28)
2nd year	0.30^**^	(0.10, 0.49)	0.04	(−0.21, 0.28)
3rd year	0.20^†^	(−0.07, 0.47)	0.16	(−0.15, 0.46)
4th year	0.08	(−0.24, 0.40)	0.16	(−0.20, 0.52)
5th year	0.15	(−0.22, 0.52)	−0.31	(−0.71, 0.08)

Doing a paid job	No		Yes	
1st year	0.16^†^	(−0.00, 0.32)	0.20^**^	(0.06, 0.34)
2nd year	0.02	(−0.22, 0.25)	0.34^**^	(0.13, 0.56)
3rd year	0.11	(−0.16, 0.38)	0.26^†^	(−0.03, 0.55)
4th year	−0.20	(−0.50, 0.11)	0.51^**^	(0.17, 0.86)
5th year	−0.33^†^	(−0.68, 0.02)	0.26	(−0.13, 0.65)

CI, confidence interval.

^a^12,554 observations of 12,554 individuals after multiple imputation.

^b^Adjusted for covariates. See Table 2 for covariates.

^***^P < 0.001, ^**^P < 0.01, ^*^P < 0.05, ^†^*P* < 0.10.

Lastly, Table 4 summarized the results of the IV models, which took the instrumented husband’s retirement age as the alternative baseline. The patterns of the results observed in Table 3 remained intact in general. The wife’s active social participation, intensive interactions with her husband, and doing no job prevented her mental health from deteriorating, while the impact of social participation was observed only in the first year.

**Table 4.** Comparing the changes in the wife’s K6 score by her baseline behavior: IV approach^a,b^

Social participation	Less active		Active	
1st year	0.28^**^	(0.08, 0.49)	0.08	(−0.22, 0.37)
2nd year	0.19	(−0.05, 0.43)	0.26	(−0.08, 0.60)
3rd year	−0.18	(−0.46, 0.11)	−0.19	(−0.58, 0.20)
4th year	0.14	(−0.21, 0.50)	0.20	(−0.30, 0.69)
5th year	0.01	(−0.43, 0.46)	−0.08	(−0.78, 0.62)

Interactions with the husband	Less intensive		Intensive	
1st year	0.28^*^	(0.04, 0.51)	0.18	(−0.10, 0.45)
2nd year	0.31^*^	(0.03, 0.59)	0.13	(−0.19, 0.44)
3rd year	−0.09	(−0.42, 0.24)	−0.20	(−0.57, 0.17)
4th year	0.39^†^	(−0.03, 0.80)	0.01	(−0.45, 0.47)
5th year	−0.01	(−0.53, 0.52)	−0.08	(−0.69, 0.53)

Doing a paid job	No		Yes	
1st year	0.09	(−0.15, 0.33)	0.30^**^	(0.07, 0.53)
2nd year	−0.01	(−0.29, 0.26)	0.37^**^	(0.10, 0.64)
3rd year	−0.20	(−0.51, 0.11)	−0.15	(−0.47, 0.17)
4th year	−0.12	(−0.53, 0.28)	0.39^†^	(−0.01, 0.78)
5th year	−0.25	(−0.82, 0.31)	0.15	(−0.34, 0.63)

^a^12,554 observations of 12,554 individuals after multiple imputation. Using public pension eligibility ages as instrumental variables for the husband’s retirement age.

^b^Adjusted for covariates. See Table 2 for covariates.

^***^P < 0.001, ^**^P < 0.01, ^*^P < 0.05, ^†^*P* < 0.10.

## DISCUSSION

We examined how a husband’s retirement affected his wife’s mental health using a population-based longitudinal survey, which could identify the timing of the husband’s retirement. Unlike most preceding studies regarding the husband retirement syndrome, this study focused on the impact of the wife’s behavior prior to the husband’s retirement on the evolution of her mental health after retirement. The key findings and their interpretations are summarized as follows.

First, our regression results confirmed the existence of the husband retirement syndrome, in line with preceding studies,^10^^,^^11^ as long as all the respondents were used in regression analysis. The wife’s mental health, measured via the K6 score, deteriorated in response to her husband’s retirement. Two points should be noted here. First, we observed the adaptation process of the wife’s mental health, as is generally observed for life events.^31^ The K6 score jumped at the husband’s retirement and remained above the pre-retirement level for a couple of years before declining, rather than continuing to climb. Second, however, the deterioration in mental health was relatively moderate; the K6 score increased by only 4–5% of its standard deviation.

More importantly, the results suggest that the husband retirement syndrome depended strongly on conditions prior to the husband’s retirement, underscoring the wife’s behavior as an effect modifier for the impact of her husband’s retirement on the evolution of her mental health. This message was confirmed even if we controlled for the endogeneity of the husband’s retirement by the IV method.

Specifically, active social participation, intense interactions with the husband, and doing no paid work are found to have prevented the wife’s mental health from deteriorating. The first two findings are consistent with previous findings that active social participation and high marital quality favorably impact health.^15^^–^^18^ The third finding suggests that wives working outside the house are more likely to feel stressed by adaptation to their husbands’ retirement,^19^^,^^20^ although we cannot exclude the opposite possibility depending on different socio-cultural contexts and other factors.

The results in this study suggest the need for policy measures to help middle-aged couples to have successful retirement life, including municipalities’ support to encourage couples to participate in various social activities and provide them with counselling services to deal with retirement-related mental health problems. Meanwhile, it should be also noted that the impact of a spouse’s retirement on health must depend greatly on gender-related social norms, which may differ across countries and change over time. Hence, further studies are required to compare the magnitude and quality of husband’s retirement syndrome across the countries and examine its long-term trends in each country. Especially in Japan, increases in dual-earner couples will them to decide their retirement more jointly, requiring a more in-depth analysis of the couple’s post-retirement well-being.

We concentrated on the wife’s behavior prior to her husband’s retirement, rather than her current behavior, in order to mitigate potential endogeneity biases. To be sure, the husband’s retirement may encourage his wife (and possibly himself as well) to participate in more social activities and share more time together with the spouse. In addition, the wife’s retirement may be jointly determined with her husband’s.^32^ Considering these possible adjustments in the wife’s behavior, the observed striking differences in the paths of the wife’s mental health by the wife’s behavior prior to the husband’s retirement were notable. The results did not identify any causation from the wife’s behavior at baseline to her psychological response to her husband’s retirement; for example, the wife’s behavior likely reflects individual attributes, such as personality traits, which were not controlled for in this study. However, we can reasonably argue that the wife’s behavior prior to her husband’s retirement was a reliable predictor of the evolution of her mental health thereafter.

We recognize that this study has several limitations and drawbacks in addition to tentative thresholds for defining active social activity and intensive interaction with the husband, as well as a lack of information about changes in the wife’s daily time use. First, we did not consider two-way spillover effects of health between the spouses,^3^ mainly due to limited information about the spouse’s behavior available from the survey. This study thus ignored the possible impact of a change in the wife’s health and/or health behavior on the retired husband’s, which in turn may affect the wife’s. This feedback mechanism is also expected to be affected by the couple’s pre-retirement behavior and marital quality.

Second, we ignored the case where a wife’s deteriorated mental state led to divorce because we did not look at the mental health of female respondents after divorce. This implies that the estimation results may underestimate the negative impact of the husband’s retirement on her wife’s mental health, even if the couple’s decision to divorce is likely affected by other spousal/family-related factors as well.

Third, we should be cautious in any generalization of these results, which were obtained from the Longitudinal Survey of Middle-Aged and Older Adults conducted in Japan. This survey focused exclusively on the behavior of the cohorts aged 50–59 years in 2005 observed during the subsequent 11 years, neglecting possible differences between cohorts. For instance, younger cohorts likely have a higher proportion of dual-earner couples, who tend to make their retirement more jointly decided, leading to a change of implications of retirement on their mental health.

Despite these caveats, the results suggest the limited relevance of the “retired husband syndrome” in middle-aged Japanese couples. We can assume that the response of the wife’s mental health to her husband’s retirement strongly depended on her behavior before it.
